# DNA Damage and Repair in Human Cancer: Molecular Mechanisms and Contribution to Therapy-Related Leukemias

**DOI:** 10.3390/ijerph9082636

**Published:** 2012-07-27

**Authors:** Ida Casorelli, Cecilia Bossa, Margherita Bignami

**Affiliations:** 1 Azienda Ospedaliera Sant’Andrea, Via di Grottarossa 1035-1039, Roma 00189, Italy; Email: icasorelli@ospedalesantandrea.it; 2 Department of Environment and Primary Prevention, Istituto Superiore di Sanità, Viale Regina Elena 299, Roma 00161, Italy; Email: cecilia.bossa@iss.it

**Keywords:** therapy-related leukemia, DNA repair, chemotherapeutic drugs

## Abstract

Most antitumour therapies damage tumour cell DNA either directly or indirectly. Without repair, damage can result in genetic instability and eventually cancer. The strong association between the lack of DNA damage repair, mutations and cancer is dramatically demonstrated by a number of cancer-prone human syndromes, such as xeroderma pigmentosum, ataxia-telangiectasia and Fanconi anemia. Notably, DNA damage responses, and particularly DNA repair, influence the outcome of therapy. Because DNA repair normally excises lethal DNA lesions, it is intuitive that efficient repair will contribute to intrinsic drug resistance. Unexpectedly, a paradoxical relationship between DNA mismatch repair and drug sensitivity has been revealed by model studies in cell lines. This suggests that connections between DNA repair mechanism efficiency and tumour therapy might be more complex. Here, we review the evidence for the contribution of carcinogenic properties of several drugs as well as of alterations in specific mechanisms involved in drug-induced DNA damage response and repair in the pathogenesis of therapy-related cancers.

## 1. Introduction

According to the World Health Organization (WHO) cancer is the second leading cause of death in developed countries. In the latest decades survival of cancer patients has improved dramatically as a result of development of new therapeutic regimes, although a parallel increase in the frequency of new malignancies among cancer survivors has been identified (for recent comprehensive reviews see references [1,2,3,4,5]. Therapy-related myeloid neoplasms (t-MN) are the most prevalent forms of secondary cancer and account for approximately 10 to 20 percent of myeloid neoplasms. This is a heterogeneous and poorly defined group of patients who include myelodysplastic syndromes (t-MDS), acute myeloid leukemia (t-AML) and myelodysplastic/myeloproliferative neoplasms (t-MDS/MPN). t-MN have been defined by the 2008 WHO classification system as clonal hematopoietic stem cell disorders related to previous exposure to chemotherapy and/or radiation therapy [6]. The characteristics of t-MN and the timing of its development after diagnosis of the primary disease depend on the exposure to specific agents as well as the cumulative dose and dose intensity of the preceding cytotoxic therapy. Antineoplastic drugs have been used in the treatment of malignant diseases for more than 50 years. There are almost 100 antineoplastic drugs currently in use, many of which are mutagenic and either known or probable human carcinogens. Depending on the chemotherapeutic agent and/or radiation, two main subtypes of t-MN have been distinguished: alkylating agent-related and topoisomerase II (topoII) inhibitor-related t-MN [7]. The most common subtype, occurring after exposure to alkylating agents and/or radiation with a latency period of 5–10 years, is frequently accompanied by unbalanced cytogenetic abnormalities, such as loss of all or parts of chromosomes 5 and/or 7. The second less common subtype, arising after treatment with agents targeting topoII, has a shorter latency period of 1–5 years and frequently exhibits balanced chromosomal rearrangements involving *MLL*, *RUNX1*, and *PML-RARA* genes. The risk associated with alkylating agent and radiation exposure appears to increase with age, while the risk associated with topoII inhibitors appears to be constant across all ages. However, because in recent years most patients received treatment with both alkylating agents and drugs that target topoII for previous malignancy, discrimination according to the type of previous therapy is often not feasible. Thus, in the current WHO classification t-MN are no longer subcategorized. t-MN have been also described following antimetabolites, such as fluorouracil, methotrexate, azathioprine and fludarabine. 

Hematopoietic progenitor cells that survive following exposure to DNA damaging agents could harbour acquired mutations caused by unrepaired or misrepaired damage and could then be at risk for leukemic transformation. The strong association between lack of DNA repair, genetic instability and cancer is dramatically demonstrated by a number of cancer-prone human syndromes, such as xeroderma pigmentosum, ataxia-telangiectasia, Fanconi anemia, Hereditary Nonpolyposis Colorectal Cancer and MUTYH-Associated Polyposis. In some instances, however, a DNA repair mechanism might become error-prone and introduce lethal damage into the cell. An example is the paradoxical relationship between DNA mismatch repair (MMR) and methylation damage sensitivity. This suggests that the connection between DNA repair efficiency and carcinogenesis might be more complex than previously envisaged. t-MN provides a unique opportunity to examine the effects of mutagens on carcinogenesis in humans, as well as the role of genetic susceptibility to cancer. Here we will discuss the type of DNA damage induced by chemotherapeutic drugs as well as the specific DNA repair pathways involved in damage removal and their possible involvement in the etiology of t-MN. 

## 2. Results and Discussion

### 2.1. t-MN Following Therapy with Alkylating Agents

Alkylating agents are a large class of chemotherapeutic drugs and play an important role in the treatment of several types of cancers. For more than two decades patients treated with alkylating agents have been identified as being at risk of developing t-MN. These were characterized by deletion or loss of the long arm of 7 and/or chromosome 5. More recently a sub-classification of genetic events occurring in these t-MN based on the presence or absence of chromosome 5 loss has been proposed [2,8]. How DNA damage and/or DNA repair induced by alkylating agents is involved in the aetiology of the t-MN? These drugs can be divided into monofunctional (e.g., temozolomide, procarbazine and dacarbazine) or bifunctional alkylating agents such as chloroethylating nitrosoureas (e.g., carmustine (BCNU), lomustine (CCNU), nimustine (ACNU)), alkylsulfonates (e.g., busulfan) and nitrogen mustards (chlorambucil, melphalan and cyclophosphamide). Both monofunctional and bifunctional alkylating agents are mutagenic and genotoxic, although the type of damage and the repair pathways acting on DNA lesions are quite different. 

Monofunctional alkylating agents can produce several adducts in DNA. 7-methylguanine (7-meG) and 3-methyladenine (3-meA) are removed via the Base Excision repair (BER) pathway, while the major miscoding and toxic lesion, O^6^-methylguanine (O^6^-meG) is repaired by the O^6^-methylguanine DNA methyltransferase (MGMT) (Table 1).

**Table 1:** Major monofunctional alkylating agents associated with the risk of occurrence of t-MN.

Monofunctional agents			DNA adduct	DNA repair
Dacarbazine	Procarbazine	Temozolomide		
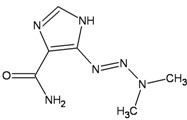	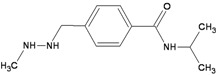	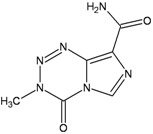	**O^6^–meG ** **→**	**MGMT, MMR**
			**DSBs ** **→**	**FANC** ** pathway (HR)**
			3–meA; 7-meG →	BER

**Figure 1:**
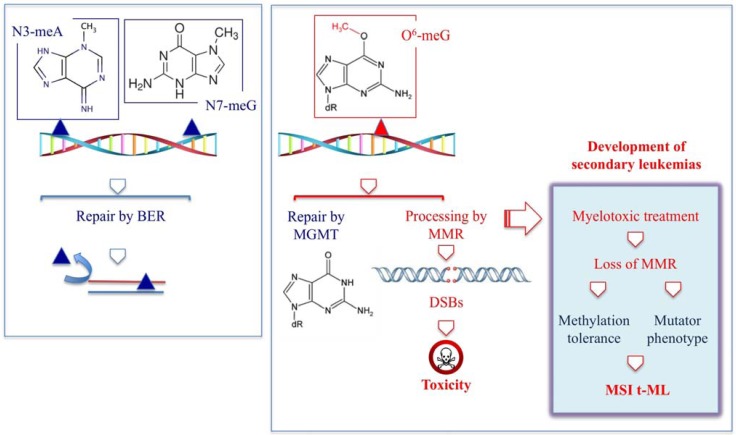
Repair and processing of DNA damage induced by monofunctional methylating agents.

Since this enzyme can be limiting in some tissues (including the bone marrow), the methylated base might persist in the genome and its ability to miscode at replication will introduce DNA mismatches which are recognized by MMR. MMR however does not remove the methylated base but processes the opposite strand and these unsuccessful repair attempts result into lethal double strand breaks (DSBs). Thus inactivation of MMR is associated with resistance to killing by methylating agents (methylation tolerant phenotype) and a striking mutator phenotype (Figure 1) [9]. 

This mechanism might underlie the selection and clonal expansion of rare MMR- defective myeloid precursor cells with a selective survival advantage in patients treated with drugs for which myelotoxicity is dose limiting [10,11]. The mutator phenotype that is characteristic of MMR deficient cells would accelerate the development of t-MN. Indeed t-AML occurring after treatment with alkylating drugs display the microsatellite instability (MSI) characteristic of MMR-defective tumors [12,13,14,15], whereas this phenotype is absent in *de novo*. This is also consistent with the observation that in the rare cases with biallelic germline mutations in MMR genes (constitutional mismatch repair-deficiency) the cancer syndrome is characterized by a spectrum of early-onset malignancies with haematological malignancies prevailing in patients with MLH1 or MSH2 mutations (for a review see reference [16]). Which are the MMR genes inactivated in t-MN and which is the underlying mechanism is unclear. Although hypermethylation of the hMLH1 promoter does not seem to be a frequent event in these malignancies [14,17], a common polymorphism at position -93 in the core promoter of MLH1 has been proposed as a risk allele for the development of cancer after methylating chemotherapy for Hodgkin lymphoma [18]. The MLH1-93 variant allele was over-represented in t-AML cases when compared to *de novo* AML cases and healthy controls and was associated with a significantly increased risk of developing t-AML but only in patients previously treated with a methylating agent. 

**Table 2:** Major bifunctional (alkylating) agents associated with the risk of occurrence of t-MN.

Drug	DNA adduct	DNA repair	References
**BCNU, CCNU, ACNU**			
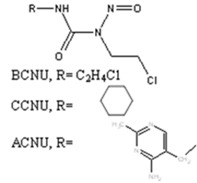	**Monoadducts N^1^G, N^7^O^6^G** **N^1^G:N^3^C interstrand CL** Intrastrand CLs DNA-protein CL	**MGMT** **FANC pathway (HR, NER)** NHEJ, BER (minor)	[19,20,21]
**Cyclophosphamide**			
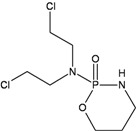	**N^7^G:N^7^G interstrand CL** N^7^G monoadducts Phosphotriester monoadducts Acrolein DNA-protein CL	**FANC pathway (HR, NER)** MGMT (?)	[22,23,24,25,26,27,28]
**Chlorambucil**			
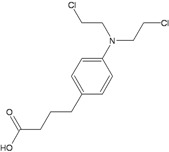	N^3^A, N^7^G monoadducts N^7^G:N^7^G and N^7^G:N^3^A (?) Interstrand and intrastrand CLs N^3^A:N^3^A intrastrand CL aneuploidy	BER **FANC pathway (NER, HR)**	[29,30,31,32]
**Melphalan**			
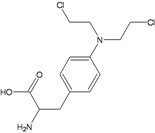	**N^3^A, N^7^G monoadducts** 5'AGT3' interstrand CL N^7^G:N^7^G and N^7^G:N^3^A (?) Interstrand and intrastrand CLs aneuploidy	**NER** **FANC pathway (NER, HR)** BER(?) NHEJ	[30,31,32,33]
**Busulfan**			
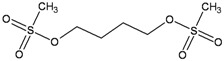	**Monoadducts N^7^G, N^3^A or N^7^A (?)** **5'GA3', 5'GG3' intrastrand CL** N^7^G:N^7^G interstrand CL (weak) DNA-protein CL	**BER** **MMR** FANC pathway (NER, HR)	[20,21,34,35,36]
**Platinum compounds**			
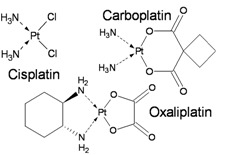	**N^7^G:N^7^G intrastrand CL 1,2-d(GpG)**, 1,3-d(GpNpG) N^7^G:N^7^A intrastrand CL 1,2-d(ApG) N^7^G:N^7^G interstrand CL	**NER** MMR **FANC pathway (NER, HR)**	[37,38,39,40,41]

Bifunctional alkylating agents possess two reactive sites and can form, in addition to monoadducts, intra- and interstrand crosslinks (CLs) by attacking two bases within the same or opposite DNA strands (Table 2). In the case of chloroethylnitrosoureas (CCNU, BCNU, ACNU) the main toxic lesions have been identified in the CLs formed between N^1^G and N^3^C following a rearrangement of the initial O^6^-(2-chloroethyl)guanine adduct to a cyclic N1, O^6^-ethanoguanine, which then reacts with the N3-position of cytosine in the opposite DNA strand. There is a general consensus that DSBs are generated as a result of collapsed replication forks encountering the CLs. Several repair systems act on chloroethylnitrosoureas-induced DNA damage. Substrates for MGMT include the O^6^-(2-chloroethyl)guanine and N^1^, O^6^-ethanoguanine, monoadducts and inactivation of the repair enzyme is consistently associated with an increased drug toxicity (for a review, see [19]). In addition the repair of the CLs requires a combination of Fanconi proteins, nucleotide excision repair (NER), translesion DNA polymerases and factors involved in homologous recombination (HR) (for a review see reference [42]). 

Nitrogen mustards are also powerful inducers of CLs, but different alkylators, although sharing the same reactive functional group, can introduce into DNA lesions which are variable in nature and proportion [29,32,43]. Thus some uncertainties persist on the identification of the major toxic and/or mutagenic lesions and the repair pathways involved in their removal (Table 2). All nitrogen mustards induce monofunctional guanine-N7 adducts, as well as interstrand N7-N7 CLs involving the two distal guanines within GNC sequences. In addition, melphalan and chlorambucil also induce substantial alkylation at adenine N^3^, including possible formation of guanine-adenine CLs [31,34]. On the other hand, the oxazaphosphorine cyclophosphamide undergoes metabolic activation into phosphoramide mustard and acrolein reactive species [22]. Phosphoramide mustard is thought to induce phosphotriester and N^7^G monoadducts and to a minor extent N^7^G:N^7^G interstrand CLs, probably the most relevant cytotoxic lesions [23]. Acrolein has also been proposed as the mutagenic/toxic metabolite of cyclophosphamide, with the human MGMT being involved in its repair [22]. There are however conflicting reports on the role of MGMT in modulating cyclophosphamide toxicity. In contrast to the *in vitro* observations, comparison of the therapeutic response of 23 tumor xenografts showed no correlation with MGMT levels [24] and MGMT deficiency in *Mgmt^−/−^* mice did not significantly alter short-term cyclophosphamide-induced toxicity or mutagenicity [25,26]. Surprisingly, *Mgmt-*deficient mice showed a reduced tumour incidence compared to wild-type mice [27]. This observation together with the demonstration that MGMT can be cross-linked to DNA by nitrogen mustards [28] suggests that DNA-protein CLs could participate in cyclophosphamide-induced carcinogenesis. MGMT does not modify significantly the toxicity and/or mutagenicity of other nitrogen mustards. 

Another example of the selectivity of DNA repair systems in modulating toxicity induced by nitrogen mustards is the involvement of NER in repairing melphalan-induced monoadducts, in addition to its role together with HR in CLs repair. These results suggest an important cytotoxic role of melphalan induced bulky adducts [33] which is not necessarily shared by other drugs.

Similarly to nitrogen mustards, the alkylsulfonate drug busulfan is reported to induce a variety of DNA adducts, such as monofunctional adducts (N^3^A and N^7^G), and CLs. However, the exact nature of DNA bis-alkylation products is still not clarified. Some experiments reported a weak induction of N^7^G:N^7^G interstrand CLs [34], whilst different experimental approaches supported the formation of 5’GA3’, 5’GG3’ (possibly N^7^A:N^7^G) intrastrand CLs [35]. Although it is unclear the extent of alkylation on the O^6^ position of G [36], the report that a methylation-tolerant glioblastoma multiforme xenograft (resistant to procarbazine and temozolomide) was also resistant to busulfan is a strong indication of MMR participating in the processing of busulfan-induced DNA damage [20]. In addition BER impairment results in hypersensitivity of human cells exposed to busulfan and BCNU but not to melphalan [21]. In conclusion multiple repair systems are operating on DNA damage induced by alkylating agents and misrepair or loss of repair of these highly mutagenic lesions might contribute to the generally recognized high risk of leukemogenesis associated with the clinical use of these drugs. Indeed a higher frequency of hypermethylation of the BRCA1 promoter region has been reported in t-AML compared to *de novo* AML (76 *vs.* 31%) suggesting a key role of BRCA1 deregulation in secondary leukaemogenesis [44]. In addition the molecular signature identified by microarray analysis which was able to separate therapy-related and *de novo* APL contained genes involved in cell cycle control and DNA repair [45]. Repair genes in particular, mostly belonging to MMR, recombinational repair and BER, were up- as well as down-regulated. More recently gene expression was analysed in CD34+ cells from patients who develop t-MDS/AML after autologous hematopoietic transplantation for Hodgkin and non-Hodgkin lymphomas with controls who did not develop the disease [46]. Genes involved in oxidative stress response, cell cycle regulation and DNA repair were identified as discriminatory genes. Thus an imbalance in some of the components of the repair pathways might be responsible for inefficient DNA repair and accumulation of mutations contributing to the development of t-MN. In addition to the acknowledged clastogenicity of these drugs, attention on their aneugenic potential possibly because of the ability to induce centrosome defects, has also been raised [47]. 

Another important group of chemotherapeutic agents is represented by platinum-based drugs such as cisplatin, carboplatin, and oxaliplatin. These compounds are effective broad-spectrum anticancer drugs widely used in the treatment of adult and pediatric cancers, especially solid tumors. While platinum compounds are not chemical alkylators, in the literature they are sometimes defined as alkylating-like agents; this to emphasize a similarity in the antitumor mechanisms of action. They act by forming DNA adducts leading to covalent intrastrand and interstrand CLs. The most prevalent form is the 1,2-intrastrand CL in which platinum is covalently bound to the N7 position of adjacent purine bases. Other platinum-DNA adducts include the 1,3-intrastrand and interstrand CLs at CpG sequences [37]. As previously discussed during replication CLs stall replication forks resulting in DSBs formation. Although both CLs and DSBs are highly cytotoxic DNA lesions, the effectiveness of platinum compound-based therapy is often limited by the acquisition of drug resistance which can be mediated by multiple factors including DNA repair efficiency. 

The mechanisms of repair of platinum-induced lesions are complex and requires a combination of DNA repair Fanconi proteins, NER, MMR, translesion DNA polymerases and factors involved in NHEJ and HR. Although several studies reported cisplatin resistance in MMR-defective cell lines, this is only a minor effect when compared to alkylating agent tolerance [38,39]. Studies from cancer cell lines and tumor tissues indicate a strong correlation between levels of some NER factors and cellular sensitivity to platinating agents [40]. In particular, cell lines that develop *in vitro* resistance following exposure to cisplatin showed increased expression of ERCC1 [41]. Thus assessment of ERCC1 mRNA expression in patient tumor tissues has been included in a cisplatin-based phase III trial for an individualized approach to therapy of non-small-cell lung cancer [48]. Finally one of the most impressive success of cisplatin-based therapy is shown by its role in treating metastatic testicular cancer which has resulted in >90% of patients achieving cure. This success, however, is offset by the emergence of considerable long-term morbidity, including second malignant neoplasms. In addition a strong dose–response relation between the cumulative amount of cisplatin and subsequent leukemia risk has been reported [49].

Whether generally acknowledged differences in leukemogenesis associated with some of these alkylating drugs are due to dissimilarities in the mutagenic potential of individual DNA lesions, inefficiency of their repair pathways or differences in the drug dosage of various clinical protocols remains to be clarified. 

### 2.2. t-MN Following Therapy with Topoisomerase Inhibitors

TopoII inhibitors are a group of natural and synthetic compounds widely employed for the clinical treatment of human malignancies. Among them, epipodophyllotoxins (e.g., etoposide), anthracyclines (e.g., doxorubicin, epirubicin), and anthracenediones (e.g., mitoxantrone) (Table 3) are used as anticancer agents.

**Table 3:** Inhibitors of topoisomerases associated with the risk of occurrence of t-MN.

Drugs	Chemical Structure	DNA adduct	DHA repair	References
***Topoisomerase II***				
**Etoposide**	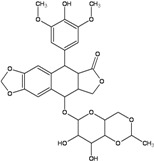	DSBs	NHEJ, HR	[50,51]
**Doxorubicin**	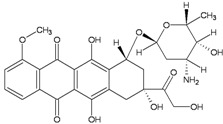	DSBs Formaldehyde-activated monoadducts at CpG (N^2^G) Oxidative damage (AP sites)	HR, NER, NHEJ, BER ?	[52,53,54]
**Epirubicin**	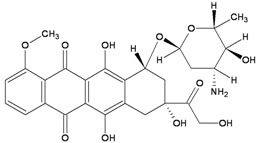	DSBs Formaldehyde-activated monoadducts at CpG (N^2^G) Oxidative damage (AP sites)	NHEJ, HR, NER, BER ?	
**Mitoxantrone**	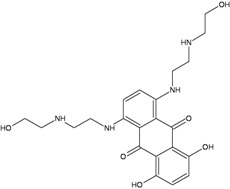	DSBs Formaldehyde-activated monoadducts at CpG (N^2^G) Oxidative damage (AP sites)	NHEJ, HR	[55]
***Topoisomerase I***				
**Camptothecin**	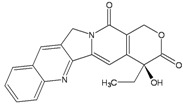	SSBs, DSBs	Tdp1, HR, NHEJ	[56]

These drugs enhance the level of topoII-DNA cleavage complexes (by inhibiting the religation reaction or by increasing the forward cleavage reaction), leading to the accumulation of DSBs. They are commonly classified as topoII poisons to distinguish them from the topoII catalytic inhibitors, which decrease the overall activity of the enzyme [51]. Although topoII poisoning is considered as a key component, other mechanisms of action have been implicated in anti-neoplastic effect of some of these drugs. In particular, anthracyclines and their synthetic analogs anthracenediones, are intercalative agents and bind DNA with very high affinities [52]. Covalent DNA adducts, preferentially at CpG and CpA sequences by mitoxantrone and (presumably) at CpG sequences by doxorubicin, have been documented [57,58,59,60]. Adducts formation is reported to be activated by formaldehyde, which contribute with its carbon atom to a N-C-N aminal linkage between the drug and a guanine residue in DNA [53,57,59]. Binding to the complementary DNA strand is through hydrogen bonding which stabilizes the drug–DNA adduct. These types of DNA adducts are repaired by pathways not completely overlapping with those operating on interstrand CLs [52,54] (Table 3). In addition, it is known that redox cycling of the quinone portion of anthracyclines leads to the production of radical species, which can directly damage DNA and result in a set of DNA lesions (e.g., AP sites) which can further contribute to poisoning of the topoII activity [61]. Indeed, it is this radical generating mechanism that is a major cause of the cardiotoxicity, which is a serious side effect of high dosages of anthracyclines [62]. Potential for cardiotoxicity has been reported for mitoxantrone also, but a different mechanism of cardiac damage induction from those of anthracyclines has been suggested [63]. Together with the ones mentioned above, other cellular responses to anthracyclines have emerged [62], nevertheless the relative contribution to cancer cell killing from these sources is still under debate. 

Camptothecins target eukaryotic type IB topoisomerase (Top1), an enzyme that cleaves one DNA strand at a time to perform its catalytic function. In spite of the fact that Top 1 is the sole target of camptothecins, the molecular determinants of their anticancer activity are complex. Drug-stabilized Top1-DNA cleavage complex are converted into DNA damage by two main processes: DNA replication and transcription. The double-strand end generated by the stalled polymerase generates an irreversible Top1-DNA crosslink associated with a double-strand end. The repair of such lesions is only partially understood, with the tyrosyl-DNA phosphodiesterase 1 (Tdp1) protein as well as proteins involved in recombinational repair playing major roles [56]. 

The association between a previous therapy with various topoII poisons and development of t-AML with different balanced translocations is well established (for comprehensive reviews see reference [1,3,64]). Translocations affecting the breakpoint cluster region of the *MLL* gene at chromosome band 11q23 are the most common molecular genetic aberrations in leukemias associated with topoII poisons. As far as the subtypes of DNA topoII inhibitors are concerned, a vast literature indicate that a previous therapy with epipodophyllotoxins (VP16) is associated with t-MN with 11q23 rearrangements, whereas other balanced aberrations [to 21q22, inv(16), t(15;17) and t(9;22)] are mostly associated with previous therapy with anthracyclines [65]. 

Because of the complication due to multiple lines of treatment and the large number of possible partners in the translocations, the role of the single chemotherapeutic agents in the rearrangements resulted more associative than causative. Convincing molecular evidence supporting the notion that t-MN are the consequence of previous exposures to chemotherapeutic drugs comes from studies on the t-APLs (Table 4). The chimeric protein which is considered the initiating event in the pathogenesis of this disease is the t(15;17)(q22;q21) translocation fusing the *PML* and the *RARA* genes. A considerable proportion of t-APL develop following exposure to the topoII poisons mitoxantrone and epirubicin for treatment of breast cancer [66,67], while more recently treatment of multiple sclerosis with mitoxantrone has also been associated with t-APL [68]. Molecular analysis of the breakpoint locations in the *PML* gene identified a 8 bp “hot-spot” site, which was demonstrated by *in vitro* assay to be a preferred site for mitoxantrone-induced topoII-dependent DNA cleavage [69,70]. In contrast to t-APL, genomic breakpoint junction regions occurring in *de novo* cases were dispersed in a wide region, with no obvious clustering. In a similar approach recurrent breakpoint clustering was identified in the *PML* and *RARA* genes in t-APL secondary to epirubicin treatment for breast cancer [71,72]. Because of the presence of small sequence microhomologies helping to align broken strands of DNA at the sites of the junction [50] non-homologous end joining (NHEJ) has been suggested as the main pathway involved in the repair of these breaks (for a review see reference [51]) Surprisingly the clustering of the breakpoint differed between epirubicin and mitoxantrone. It is possible that the two topoII poisons, which share the anthracenedione moiety and maintain the typical planar ring structure that allows intercalation between DNA base pairs, might differ in their ability to induce oxidative stress and/or in the sequence specificity of DNA adduction [55,72].

**Table 4:** Therapy-related APL and drugs for treating primary cancer.

No patients	Primary cancer /disease	Drugs	References
2 t-APL	seminoma, breast cancer	Etoposide, cisplatin, bleomycin; 4-epi-doxorubicin, cyclophosphamide, methotrexate, 5-fluorouracil, RT	[73]
106 t-APL	60 breast carcinoma; 15 non-Hodgkin’s lymphoma; 4 other hematologic; malignancies; 25 various solid tumors; 1 multiple sclerosis; 1chronic poly-radiculoneuritis	RT, RT+CT; *Alkylating agents* (cyclophosphamide, ifosfamide, chlorambucil, dacarbazine, melphalan, CCNU); *topoII inhibitors* (Doxorubicin, epirubicin, daunorubicin, mitoxantrone, VP16, VM26); *antimetabolites* (5-FU, methotrexate, cytarabine), vincristine, bleomycin, cisplatin,	[67]
17 t-APL	Langerhans cell histiocytosis	Etoposide	[74]
6 t-APL *vs.* 35 *de novo* APL	Breast cancer, multiple sclerosis	Mitoxantrone	[69]
11 t-MN *vs.* 10 relapse	108 APL	ATRA+ consolidation therapy (VP16, mitoxantrone, etoposide, daunorubicin, idarubicin, methotrexate, prednisolone)	[75]
17 t-MN	918 APL	ATRA+ consolidation therapy; (idarubicin, mitoxantrone)	[76]

Recently the development of t-MN is being reported with increasing frequency in patients successfully treated for APL [75,76] (Table 4). These *PML-RARA*-negative t-MN display specific *RUNX1* gene mutations which might also derive from genotoxic events induced by courses of consolidation chemotherapy containing topoII inhibitors.

Intriguingly, bioflavonoids, topoII inhibitors which occur naturally in food, have been reported to cause site-specific DNA cleavage in the MLL breakpoint cluster region. These colocalize with topoII cleavage sites induced by etoposide and doxorubicin. Thus it has been suggested that maternal ingestion of bioflavonoids may induce MLL breaks and potentially translocations in utero leading to infant and early childhood leukemia [77]. 

In conclusion these data strongly support the hypothesis that leukemia-associated chromosomal translocations derive from misrepair of chemically-induced DSBs occurring in susceptible regions of the genome. 

### 2.3. Antimetabolites

Antimetabolites account for nearly 1/5th of all drugs currently approved by the FDA for the treatment of cancer. These compounds, which are structural analogs of natural compounds, are used primarily in the treatment of hematological malignancies, although some of the more recently developed agents have demonstrated activity against solid tumors. The majority of antimetabolites are analogs of purines or pyrimidines and must be activated by cellular enzymes to nucleotide metabolites, which are incorporated into DNA and/or are direct inhibitors of enzymes required for DNA synthesis, such as DNA polymerases or thymidylate synthase (Table 5).

**Table 5:** Antimetabolites associated with the risk of occurrence of t-M.

	Chemical structure	DHA adduct	DNA repair
**5-Fluorouracil**	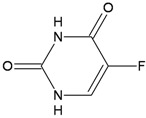	**DNA 5-FU:G mismatches** **DNA 5-FU:A mismatches** **RNA FU containing mismatches**	**BER (TGD, MBD4, SMUG, UNG)** **MMR**
**Fludarabine**	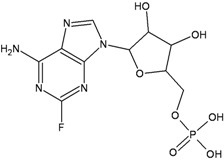	**DSBs** **F-Ara-A:T mismatches**	**NHEJ** **BER (UNG)** **Inhibition NER during ICLs repair**
**Azathioprine**	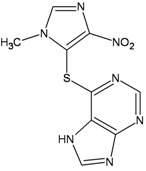	**6-TG:A mismatches**	**MMR** BER

5-Fluorouracil (5-FU) and its deoxynucleoside, 5-fluoro-2'-deoxyuridine (5-FdUrd), are the most commonly used drugs for colorectal cancer therapy. The mechanism underlying the therapeutic effect of 5-FU is complex, and precisely how 5-FU kills cancerous cells is not well understood. Intracellular metabolites of 5-FU can exert cytotoxic effects through incorporation into RNA and/or DNA. Cytotoxicity has been linked to 5-fluorouridine monophosphate (5-FUMP) incorporation into RNA and to inhibition of DNA synthesis—either directly following 5-fluoro-deoxyuridine-monophosphate (FdUMP) incorporation, or via inhibition of thymidylate synthase leading to depletion of the deoxythymidine triphosphate pool. Several DNA repair pathways can excise 5-FU adducts from DNA. Since 5-FU is a uracil analog, uracil-DNA glycosylase (UNG), single strand–selective monofunctional uracil-DNA glycosylase 1 (SMUG1), thymine DNA glycosylase (TDG) and methyl-CpG binding domain protein 4 (MBD4) are all BER enzymes capable of processing 5-FU in DNA, albeit with different kinetic properties and variable consequences for toxicity. For example, TDG or MBD4 knockout MEFs display 5-FU tolerance [78,79], Smug^−/−^ MEFs are hypersensensitive to 5-FU killing [80] and the response of Ung^−/−^ MEFs is essentially identical to wild-type cells [81]. The contribution of MMR is limited to 5-FU:G contexts [82] consistently with the role of this repair pathway in detecting a DNA mismatch (analogous to a U:G mispair). However long exposures to fluoropyrimidines might result in unbalanced nucleotide pools and in these specific conditions repair tracts produced by MMR might become cytotoxic [83]. Repair polymerases would be unable to replace FdUMP residues with normal dTMP or dCMP and would reincorporate FdUMP into DNA, triggering rounds of futile repair cycles. The futile attempts of MMR (or BER) to remove FU residues from DNA will then expose single-stranded regions and give rise to lethal DSBs. Finally, despite extensive investigations on the relative contribution of MMR and the single BER enzymes in the repair of fluoropyrimidines, whether the major effects on cell killing depend on 5-FU incorporation into RNA is still a matter of debate [81].

Fludarabine monotherapy is an established regimen for treatment of chronic lymphocytic leukemia. Recently it has been shown that the incidence of t-MN was increased by the combined exposure to cyclophosphamide-fludarabine *versus* fludarabine alone [84]. The purine analogue 9-β−D-Arabinofuranosyl-2-fluoroadenine can be converted by the cells into its triphosphate (F-ara-ATP) and incorporated into DNA, leading to termination of DNA synthesis at incorporated sites on the daughter strand [85]. This inhibition of DNA replication results in DSBs in early S phase which are mainly processed by NHEJ [86]. In addition F-ara-ATP, the main metabolite of fludarabine, can also inhibit ribonucleotide reductase with a reduction and imbalance of deoxynucleotide pools. This imbalance might favor the incorporation of F-ara-A and other mismatched nucleotides such as uridine. These mismatched base pairs would activate BER, with UDG having an important role in the removal of incorporated fludarabine from DNA [87]. It has also been proposed that the synergistic cytotoxicity of F-ara-A and agents that introduce interstrand CLs might depend on the inhibition of NER during their repair [88,89]. It is tempting to speculate that this interference might underlay the presence of misrepaired damage contributing to the accumulation of mutations/rearrangements selected during the process of leukemogenesis. 

Azathioprine is a thiopurine prodrug that requires enzymatic conversion for clinical effectiveness. The thiopurines 6-mercaptopurine (6-MP), azathioprine and 6-thioguanine (6-TG) are powerful anticancer, anti-inflammatory and immunosuppressant drugs. Despite their decades-long clinical use, the mechanisms by which thiopurines are cytotoxic to cancer or immune cells remain unclear. The first step of azathioprine metabolism generates 6-MP by removal of the protecting imidazole ring. Thiopurines metabolism culminates in the incorporation of 6-TG into nucleic acids, predominantly DNA. DNA 6-TG sulphur atom is highly reactive and is a target for methylation by S-adenosylmethionine, the ubiquitous source of intracellular methyl groups for enzymatic reactions. Methylated DNA 6-TG (6-meTG) can miscode during replication to generate imperfect base pairs that recruit the MMR machinery to a potentially lethal intervention [90]. Because MMR-dependent processing is linked to apoptosis, inactivation of repair provides an escape from thiopurine-induced cell death. Thus, MMR-deficient cells can tolerate 6-meTG in their DNA, and their resistance to killing by 6-TG is well documented. MMR performs similar lethal processing on the structurally related DNA base, O^6^-meG, that is produced when cells are treated with alkylating agents. Similarly to t-MN arising after methylating agents, a high frequency of the characteristic MSI phenotype of MMR-defective cells was reported in AML/MDS occurred in recipients of organ transplants treated by Azathioprine [91]. 

Although MMR-deficient cells are substantially resistant to 6-TG, these cells are killed by high 6-TG concentrations indicating the existence of MMR-independent pathways of cell death. Recent works have shown that 6-TG is highly susceptible to oxidation, particularly to photochemical oxidation by UVA [92]. The Azathioprine and UVA light generate mutagenic oxidative DNA damage that include bulky charged oxidized forms of DNA 6-TG, DNA breaks, DNA interstrand ICL and covalent DNA-protein adducts [93]. This photochemical damage is mutagenic and extremely toxic to cultured human cells and it has been suggested that it might contribute to the toxic effect of thiopurine/UVA treatment *in vitro* and to the high risk of skin cancer in thiopurine-treated patients. Thus both epidemiological and molecular evidence highlights an interaction between thiopurines and sunlight that may contribute to the extremely high risk of skin cancer in patients taking these drugs [94]. Similarly to t-MN, the increased risk of skin cancer, principally squamous and basal cell carcinomas in immunosuppressed organ transplant patients represents another example of therapy-related cancer [95].

### 2.4. Occupational Exposure to Chemotherapeutic Drugs

In addition to patients with documented exposure to chemotherapeutic drugs, other groups could experience perhaps less dramatic but still noteworthy, indefinite environmental or occupational contacts with mutagenic agents. Here we provide a short survey of epidemiologic studies of genotoxic/cancer risk in nurses potentially exposed to antineoplastic drugs. Exposure to cytostatic agents is a major occupational concern in oncologic personnel since nurses may be subject to unexpected events of exposure due to the intensive contact with patients. These might occur through inhalation of aerosolized drugs, absorption *via* skin contact, contaminated intravenous tubing, disposal of equipment, or when handling patients’ excreta. The original reports of increased sister chromatid exchanges (SCE) in lymphocytes of oncology nurses and mutagenicity of their urine indicate that these health-care workers were exposed to cytotoxic drugs [96,97]. Although the levels of SCE have been found to be 15 times lower than those of patients receiving treatment, nurses might still be at risk of an occupational exposure because of low but cumulative doses of cytotoxic drugs. The introduction of protective measures led to beneficial safety improvements over the years. However the observation that small, but statistically significant genotoxic burden can be still observed in oncologic nurses emphasizes the need for a continuing effort to eliminate residual occupational risks [98,99,100].

Whether an elevated risk of cancer is associated to the mutagenic effects of the antineoplastic drugs has been investigated by several studies with contrasting results. A marginally increased risk of leukemia (RR 10.65, 95%CI 1.29–38.5) based on only two cases was suggested in a study of nurses who handled antineoplastic drugs [101]. However a large study on cancer incidence in registered nurses (n = 56,213) failed to show a statistically significant increased risk of leukemia in nurses potentially exposed to antineoplastic drugs [102].

## 3. Conclusions

t-MNs are acknowledged as severe long-term consequences of cytotoxic therapies for a primary disorder. Since the advent of more effective antineoplastic therapeutic regimes has improved the survival of cancer patients, the number of subjects with t-MNs is expected to rise. Cancers survivors represent a heterogeneous group of patients exposed to a wide range of different anticancer agents and doses. Many complex factors influence the risk of second malignancies after cancer treatment, including the chemotherapeutic protocol, the doses, the extent of damage and repair as well as the age and lifestyle choices. Similarly, individual genetic differences are increasingly being understood to play a role in how our bodies cope with and metabolize chemical toxins. Because DNA damage underlies the therapeutic mechanism of many antineoplastic drugs, DNA repair is a significant determinant of risk for therapy-related cancers. On the other hand, since only a small percentage of patients exposed to cytotoxic therapy develop t-MN it has been suggested that genetic predisposition to second cancers might be associated with polymorphisms in genes involved in drug detoxification, in the DNA damage response and/or DNA repair. Indeed, several constitutional variants in genes involved in major human DNA repair pathways have been reported in patients with t-MNs. Notably, individuals with allelic variants in MMR genes [18] or in the MDM2 and TP53 DNA damage response genes were reported to be at significantly increased risk for chemotherapy-related AML [103].

A comparison of the metabolomes of peripheral blood stem cell samples from patients who did or did not develop t-MDS/AML after undergoing haematopoietic transplantation for HL and NHL identified a series of biomarkers that discriminate patients that are predisposed to the development of t-MDS/AML [104]. The identifed dysfunctional metabolic pathways included alanine and aspartate metabolism, glyoxylate and dicarboxylate metabolism, phenylalanine metabolism, citrate acid cycle, and aminoacyl-t-RNA biosynthesis. Dysfunction in these pathways suggests a mitochondrial dysfunction that would result in a decreased ability to detoxify reactive oxygen species generated by chemo and radiation therapy leading to cancer-causing mutations. Microarray analysis carried out specifically in the CD34+ hematopoietic stem cells from the same set of patients supported this conclusion and a 38-gene signature that could distinguish patients who developed t-MDS/AML post-transplantation from those who did not was identified. Thus these changes may represent factors predisposing to risk of t-MDS/AML and/or effects of pre-transplantation therapeutic exposures [46].

The hypothesis of the occurrence of genetic predisposing factors to the development of multiple cancers and the theory of selection of unrepaired damaged cells induced by cytotoxic drugs are not necessarily mutually exclusive, but might be independent events in different subgroups of patients and/or cooperate in the development of t-MN. For instance, effects of certain polymorphisms of DNA repair genes might become apparent only in the presence of DNA-damaging agents. Our hope is that a consolidation and analysis of current epidemiological results, combined with advances in understanding molecular mechanisms of DNA damage processing, will help to elucidate the connection between therapy-related cancer risk and DNA repair.
